# Immunomodulatory effect of hypertonic saline in hemorrhagic shock

**DOI:** 10.1186/s40199-015-0130-9

**Published:** 2015-10-05

**Authors:** Javad Motaharinia, Farhad Etezadi, Azadeh Moghaddas, Mojtaba Mojtahedzadeh

**Affiliations:** Department of Pharmacotherapy, Faculty of Pharmacy, Tehran University of Medical Sciences, 16 Azar Ave, Enghelab Sq, Tehran, Iran; Department of Anesthesiology & Critical Care, Sina Hospital, Tehran University of Medical Sciences, Tehran, Iran

**Keywords:** Hypertonic saline, Hemorrhagic shock, Trauma, Anti-inflammatory, Multiple organ failure, Acute lung injury

## Abstract

Multiple organ dysfunction syndrome (MODS) and nosocomial infection following trauma-hemorrhage are among the most important causes of mortality in hemorrhagic shock patients. Dysregulation of the immune system plays a central role in MODS and a fluid having an immunomodulatory effect could be advantageous in hemorrhagic shock resuscitation. Hypertonic saline (HS) is widely used as a resuscitation fluid in trauma-hemorrhagic patients. Besides having beneficial effects on the hemodynamic parameters, HS has modulatory effects on various functions of immune cells such as degranulation, adhesion molecules and cytokines expression, as well as reactive oxygen species production. This article reviews clinical evidence for decreased organ failure and mortality in hemorrhagic shock patients resuscitated with HS. Despite promising results in animal models, results from pre-hospital and emergency department administration in human studies did not show improvement in survival, organ failure, or a reduction in nosocomial infection by HS resuscitation. Further post hoc analysis showed some benefit from HS resuscitation for severely-injured patients, those who received more than ten units of blood by transfusion, patients who underwent surgery, and victims of traumatic brain injury. Several reasons are suggested to explain the differences between clinical and animal models.

## Introduction

Multiple organ dysfunction syndrome (MODS) and nosocomial infection following trauma-hemorrhage are the most common causes of mortality in hemorrhagic shock patients [1]. The most important pathogenic mechanism underlying MODS is disproportionate excitation and dysregulation of systemic inflammatory response triggered by injury and microbial invasion. Although fluid resuscitation is the mainstay of therapy in hemorrhagic shock, reperfusion of ischemic tissues produces additional injury known as ischemia reperfusion injury (IRI) [2].

Hypertonic saline (HS) now is widely used as a resuscitation fluid during critical illness because of its beneficial hemodynamic properties, such as rapid expansion of intravascular volume, reduction of endothelial and tissue edema that improves microcirculation, improvement of blood viscosity caused by hemodilution, and increased myocardial contractility [3, 4].

Besides the improvement in hemodynamic parameters, studies on hemorrhagic shock models have shown that HS can reduce organ failure [5–8]. Recently, new findings have suggested that HS modulates local and systemic inflammatory response [9–16]. It is demonstrated that an increase of 10 to 20 mOsm/kg in plasma osmolality caused by HS can affect some functions of immune cells such as degranulation [10, 14], reactive oxygen species (ROS) production [10, 14, 17], adhesion molecules expression [15], cytokine production [18, 19], and phagocytic ability [12]. However, recent studies showed that some immunomodulatory effects of HS such as inhibition of b2 integrin expression on neutrophil surface and alteration in inflammatory cytokine production mediated via sodium- or chloride-dependent events rather than by its osmolality [16, 20].

Activation of the innate immune system and inflammatory responses are associated with IRI [2, 21]. The beneficial effects of HS can be partly explained by its ability to suppress the various functions of neutrophil including adhesion molecule expression, release of proteolytic enzymes, and production of ROS [10, 13]. Endothelial cells (ECs) activation increases the adherence of neutrophils promoting capillary congestion and the no-reflow phenomenon [22]. By inhibiting the Intercellular adhesion molecule-1 and b2 integrin upregulation, HS decreases neutrophil rolling and adherence to ECs, thereby improving microcirculation and vascular permeability [23–25]. Reactive oxygen species production during reperfusion of ischemic tissues is thought to be the main reason for uncontrolled oxidative stress [21]. Several models of hemorrhagic shock and IRI demonstrated that HS attenuates oxidative stress by decreasing inducible nitric oxide synthase expression and increasing heme oxygenase −1 expression [26–28].

Although the beneficial effects of HS and its immunomodulatory mechanisms have been demonstrated in experimental studies of hemorrhagic shock, resuscitation with HS has failed to improve patient outcomes in human studies. Several meta-analyses and review articles have concluded that there is insufficient evidence for improved survival of hemorrhagic shock patients resuscitated using HS; however, these are dated [29–31]. More recent and larger clinical studies have been performed and, as for the earlier clinical studies, these trials have provided conflicting results.

This article reviews clinical evidence for decreased organ failure and mortality in hemorrhagic shock patients resuscitated with HS, and proposes reasons for the discrepancy between results of experimental studies and clinical trials.

## Methods

Materials for this review were obtained by searching PubMed, CINAHL, Scopus, Cochrane Central Register of Controlled Trials, and Cochrane Database of Systematic Reviews. Keywords used as search terms were hypertonic saline, hypertonic solution, hypertonic NaCl, hemorrhagic shock, trauma, inflammation, anti-inflammatory, multiple organ failure syndrome, lung injury, and mortality. The search was limited to publications from 1990 to the present.

Randomized controlled trials that compared HS with or without dextran to isotonic crystalloid solutions were assessed for the study endpoints of patient survival and organ failure. Papers that assessed HS resuscitation for concomitant hemorrhagic shock and traumatic brain injury (TBI) were excluded. Only English language articles were assessed in this review.

### HS resuscitation in clinical studies of hemorrhagic shock

To date, several randomized clinical trials (RCT) have been conducted on hemorrhagic shock. Only 11 RCTs met the criteria mentioned in the method [32–42]. In most, trauma patients with hypotension were included and randomly allocated for treatment with hypertonic solutions (7.5 % HS (HS7.5 %) and dextran 70 (HSD) or isotonic crystalloid solutions in pre-hospital settings or in emergency departments. All trials were prospective and double-blind and subjects received the same 250 ml dose of either HS or isotonic crystalloid solution. Resuscitation was continued by isotonic crystalloid solutions when needed.

Four of the 11 trials were designed for emergency departments [33, 34, 37, 40], the others for pre-hospital settings. In six trials only HSD was used as the HS [32, 33, 35, 36, 40, 41]; in the others, both HS and HSD were applied [34, 37–39, 42]. Unfortunately, most of these studies were conducted prior to 2000; hence, authors assessed only early mortality and hemodynamic variables as end points and did not report secondary endpoints such as the development of acute respiratory distress syndrome (ARDS) or MODS. These studies also did not report the pretreatment acid–base status of the patients. Early and late mortality as primary outcomes and MODS and infection as secondary outcomes were reported in only four studies [35, 40–42].

Table 1 shows that, in the 11 trials assessing more than 2530 cases, results showed that a small volume of HSD or HS improved hemodynamic variables such as blood pressure and cardiac output. Importantly, nearly all confirmed that the use of HSD or HS was safe and effective for trauma patients.

**Table 1:** Prospective double-blinded randomized clinical trials on HS or HSD resuscitation in hemorrhagic shock patients

Study	Population	Resuscitation fluid	End point	Results
[32]	20 pre-hospital trauma patients with SBP ≤ 100 mmHg	HSD or LR	Survival to hospital discharge and hemodynamic variables	Improved SBP and overall survival rate.
[33]	32 trauma patients with a SBP < 80 mm Hg admitted to ED	HSD or LR	Survival to hospital discharge and hemodynamic variables	There were no differences in survival rate.
[34]	106 trauma patients with SBP <80 mm Hg for 6 % HSD or < 90 mmHg for HS and were 18 years or older admitted to ED	HS or HSD or LR	Survival to hospital discharge and hemodynamic variables	There were no differences in overall survival between any of the groups.
[35]	422 pre-hospital trauma patients ≥ 16 years with SBP ≤ 90 mmHg 72 % of participants had sustained penetrating trauma	HSD or LR	Primary end points included: survival at 24 h and 30 days (if possible). Secondary end points included: complications and safety of HSD	In the HSD 6 % group which requiring surgery: there was a significant treatment effect in favor of HSD 6 % (*p* = 0.02). This effect was significant in those patients sustaining penetrating trauma (*p* = 0.01), but not in those with blunt trauma.
[36]	166 pre-hospital trauma patients with SBP ≤ 90 mmHg	HSD or LR	Survival to hospital discharge and hemodynamic variables	There was no difference in overall survival and there is a trend to improve survival in patients with severe head injuries.
[37]	105 trauma patients ≥ 18 years with SBP < 80 mm Hg admitted to ED	HSD or HS or NS	Survival to hospital discharge, hemodynamic variables	There were no significant differences in overall complication and mortality rates in the three groups.
[38]	194 pre-hospital trauma patients with SBP < 90 mm	HSD or HS or LR	Survival to hospital discharge, hemodynamics variables, MTOS and neurological outcome scores	Overall survival in the four treatment groups was not statistically significant. Survival in the hypertonic group, however, was significantly higher than that predicted by the MTOS norms. The survival rate in the HS group was higher than that in the LR group for the cohort with baseline Glasgow Coma Scale scores of 8 or less (*P* < .05 by logistic regression and *P* < .01 by Cox proportional-hazards analysis)
[39]	258 pre-hospital trauma patients with SBP < 90 mm Hg.	HSD or HS or NS	Survival to hospital discharge, hemodynamics variables, MTOS and neurological outcome scores	There were no differences in overall survival. Improved survival vs. predicted MTOS in high-risk HS & HSD 6 % patients, HS patient with GCS 8 or less and HSD 6 % patients with unobtainable BP at the time of randomization.
[40]	212 hypovolemic shock patients admitted to ED	HSD or NS	Survival at 24 h and 30 days and complications	The 24 h survival rate was significantly higher in HSD 6 % (87 %) compared with NS (72 %) (*P* < .007). HSD 6 % improved long term survival rate significantly only in the patients with MAP < 70 mmHg (*p* < .01).
[41]	209 pre-hospital blunt trauma patients with SBP ≤90 mm Hg “The study was stopped for futility after the second interim analyses.”	HSD or LR	Primary outcome was 28 day ARDS-free survival. Secondary outcome; nosocomial infection, multiple organ failure syndrome	There was no significant difference in ARDS-free survival. There was an improved in ARDS-free survival in the patients (19 % of the population) requiring 10 U or more of packed RBC in the first 24 h. (HR, 2.18; 95 % CI, 1.09–4.36).
[42] RCT, multi center	853 pre-hospital hypovolemic shock patients with SBP ≤ 70 mm Hg or SBP ≈ 71–90 mm Hg with HR equal or higher than 108 beats per minute. (62 % of patients were with blunt trauma.) . “The study was stopped early (23 % of proposed sample size) for futility and potential safety concern.”	HSD or HS or NS	Primary outcome was 28 day survival. Secondary outcomes included: fluid and blood requirements in the first 24 h, physiologic parameters of organ dysfunction, 28 day ARDS–free survival, multiple organ dysfunction score and nosocomial infections	There was no significant difference in 28 day survival between treatment groups. There was a higher mortality for the post-randomization subgroup of patients who did not receive blood transfusions in the first 24 h, who received hypertonic fluids compared to NS (*P* < 0.01). There were no differences between groups in organ failure or nosocomial infections.

*HSD* dextran 70 in HS. *HS* hypertonic saline 7.5 %. *NS* 0.9 % saline. *LR* ringer’s lactate. *SBP* systolic blood pressure. *MAP* mean arterial pressure. *MTOS* major trauma outcome study. *BP* blood pressure. *RTS* revised trauma score. *ARDS* acute respiratory distress syndrome. *CI* confidence interval. *HR* hazard ratio. *RBC* red blood cells. *HR* heart rate. *RCT* randomized clinical study. *ED* emergency department

Results from evaluating trials were not able to show any survival improvement; only Younes et al. demonstrated in 1997 that HSD treatment significantly improved survival after 24 h of resuscitation [40]. In other studies, *post hoc* sub-group analysis suggests that certain populations could be more likely to benefit from HS; these included patients with TBI, with low mean arterial pressure (MAP), who required massive transfusions, or underwent surgery.

Younes et al. studied 212 hypovolemic shock patients in an emergency department. Patients were randomly assigned for treatment with a 250 ml bolus of 7.5 % HS + 6 % dextran (HSD, *n* = 101) or 0.9 % normal saline (NS, *n* = 111) and resuscitation was followed using a standard algorithm [40]. The groups were assessed at 24 h and 30 days of survival and prognostic factors such as sex, age, cause of hypovolemia induction, revised trauma score, Glasgow index, and MAP on admission were evaluated. The results showed that the 24 h survival rate was significantly higher for the HSD group (87 %) than the NS group (72 %) (*p* ≤ 0.007). Multivariate analysis showed that RTS and MAP were independent predictors for 24 h survival for the HSD group. HSD improved the long-term survival rate significantly only in the patients with MAP values of <70 mmHg (*p* < .01). The overall rate of complications (renal failure, ARDS, heart failure, infections, and neurological complications) were similar for both groups (24 %). The authors concluded that the administration of HS as an initial treatment of hypovolemic patients admitted through the emergency department is safe and associated with a beneficial outcomes. Further study is needed to identify the survival benefits in patients treated with HS in a pre-hospital setting.

Vassar et al. could not prove differences in survival between differently resuscitated patients in a pre-hospital setting [38]. They resuscitated injured patients with a systolic blood pressure (SBP) of <90 mmHg using four solutions (*n* = 150 in each group) containing 250 ml of lactated Ringer’s (LR), 7.5 % HS, 7.5 % sodium chloride + 6 % dextran 70, or 7.5 % sodium chloride + 12 % dextran 70 followed by conventional isotonic solutions as needed. There was no overall difference in survival between groups, but actual survival in the hypertonic group was significantly higher than that predicted by Major trauma outcome study (MTOS) norms. The survival rate in the HS group was higher than that in the LR group for patients with baseline Glasgow Coma Scale scores of ≤ 8 (*p* < 0.05 by logistic regression; *p* < 0.01 by Cox proportional hazards analysis). The authors concluded that infusion of a small volume of 7.5 % HS early in resuscitation was safe and improved the survival rates of severely-injured patients when compared with MTOS norms. The addition of dextran to hypertonic solution showed no benefits.

Prior this study, in 1991, Vassar et al. published the results of 166 pre-hospital trauma patients recording SBP ≤ 90 mmHg [36]. They resuscitated patients with 250 ml of either 7.5 % HSD (*n* = 83) or LR solution (*n* = 83). Although the results of this study showed no difference in overall survival between HSD and LR patients, it did show a trend of improved survival in patients with severe head injuries. Administration of small volumes of HSD before hospitalization increased the blood pressure of severely injured patients more effectively than did LR solution and tended to improve survival in patients with severe head injuries.

Mattox et al. randomly assigned hypovolemic shock patients to be administered with HSD or isotonic crystalloid solution (LR, NS, plasmalyte) [35]. Survival at 24 h and 30 days (if possible) were primary end points and improvement in 24 h physiological status, reduction in post-injury complications, and safety of HSD solutions (in the volume given) with regard to seizures, anaphylactoid reactions, and coagulopathies were secondary end points. The authors estimated that 700 patients would be required to show a difference in survival, but only 422 patients were enrolled. Seventy-two percent of participants had sustained penetrating trauma. HSD has improved survival significantly in the subpopulation of patients requiring surgery (*p* = 0.02). This effect was significant in those patients sustaining penetrating trauma (*p* = 0.01), but not in those with blunt trauma. Only 22 patients were followed for as long as 30 days. Thus, analysis was not performed.

The HSD group had fewer complications such as ARDS, renal failure, sepsis, pneumonia, and coagulopathy than the standard treatment group (7 versus 24). HSD related coagulopathy, anaphylactoid reactions, and seizures were not reported. The authors concluded that bolus administration of 250 ml of 7.5 % HSD is safe and was as effective as standard resuscitation solutions in the pre-hospital management of traumatic hypotension. HSD may also offer a potential benefit in a subgroup of patients with penetrating injuries, active hemorrhage, or those requiring urgent laparotomy or thoracotomy. Studies with larger sample sizes will be required to establish which subgroups of trauma patients will maximally benefit from pre-hospital use of a small volume of HS [35].

Lack of conclusive evidence led to meta-analysis to reevaluate the effect of HSD on early mortality (24 h survival). The first was performed by Wade et al. in 1997. They included eight RCT of trauma patients treated with HSD until that date and demonstrated that HSD administrated as the initial fluid therapy enhanced survival of patients with hypotension caused by trauma (OR = 1.46; 95 % CL; 1.01–2.12). All included studies in this meta-analysis have been summarized in the present article. Further improvement in survival was found in patients with penetrating trauma requiring surgery (OR = 1.97; 1.07–3.61) or blood transfusion. The authors concluded that initial fluid therapy with HSD was beneficial in patients requiring blood transfusion, surgery, or patients with penetrating injuries who required surgery [29].

The second meta-analysis examined eight double-blind RCTs in which HSD and HS were compared with isotonic solution [32–39] and also compared HS and isotonic solution in two trials for the treatment of hypovolemic trauma patients (These studies was not reviewed in the present article due to unpublished data). Analysis was done on 615 patients who were treated with 6 % HSD and 340 who were treated with HS. The primary end point was 30 day survival after injury or until discharge. Results showed that the survival rate did not differ for HS and isotonic resuscitation (*p* = 0.46; OR = 0.98; 95 % CI; 0.71–1.36). The authors concluded that there is a trend toward superiority of 6 % HSD for improved survival, but it was not significant. The results of the meta-analysis showed that resuscitation with HS alone did not demonstrate efficacy for survival of trauma patients with hypovolemia [30].

Bunn et al. conducted meta-analysis in 2004 to determine whether HS decreases mortality in hypovolemic patients with and without head injury [31]. Most of the 17 trials (869 participants) included in the analysis had small precipitants and only five trials conducted on trauma patients were judged to provide adequate quality [32–39] (Nine of the studies that have been included in this meta-analysis was not reviewed in the present article due to unpublished data and included TBI patients). The authors concluded that there was insufficient data to support efficacy of HS in resuscitation of patients with trauma, burns, or those who underwent surgery. They stated that further large and qualified trials were needed to adequately compare hypertonic and isotonic crystalloid.

Bulgar et al. in 2008 designed a RCT to evaluate effect of HS on late mortality, multiple organ failure, and nosocomial infection in blunt traumatic injury with hypovolemic shock. In this study, 209 patients with SBP of ≤ 90 mmHg were randomized to receive either HSD (*n* = 110) or LR (*n* = 99) in a pre-hospital setting. The primary outcome was 28 day ARDS-free survival and the secondary outcomes were nosocomial infection, MODS, resource utilization, mortality, and noninfectious complications. There was no significant difference in ARDS-free survival (adjusted hazard ratio = 1.01; 95 % confidence interval (CI); 0.63–1.60); however, there was evidence of improvement in ARDS-free survival by patients (19 % of the population) requiring 10 units or more of packed red blood cells in the first 24 h. (Odds ratio (OR) = 2.18; 95 % CI; 1.09–4.36). Further evaluation using Cox proportional hazards methods to assess the effect of treatment by red cells transfused again reached the same hazard ratio for 28 day survival (2.49; 95 % CI; 1.1–5.6). There were no significant differences in the secondary outcome measures. The authors concluded that although there were no significant differences in ARDS-free survival and secondary outcomes, there was a survival benefit in the subgroup of patients at highest risk for ARDS, such as those requiring 10 units or more of packed red blood cells in the first 24 h [41].

Bulger et al. in 2011 conducted a larger trial on traumatic hemorrhagic shock in patients. The resuscitation outcome consortium (ROC) trial was a randomized, double-blind, multicenter study. Two series of patients were included; those who recorded SBP ≤ 70 mmHg and those with heart rates (HR) of ≤ 108 beats per minute and SBP of 71 to 90 mmHg. Patients randomly received a 250 ml bolus of HSD, HS, or NS followed by additional crystalloid as determined by medical requirements in a pre-hospital setting. The primary outcome was 28 day survival and the secondary outcomes were incidence of ARDS, multi-organ failure, infection, number of ventilator days, and physiological and functional outcomes in the first 28 days after intervention. Because only 23 % of the proposed sample size was achieved and because of the increase in mortality among patients receiving no blood transfusions in the first 24 h, the Data Safety Monitoring Board halted the study prematurely. The 28 day mortality rates did not differ between HS, HSD and NS groups (74.5 % HSD (absolute difference in 28 day survival probabilities = 0.1; 95 % CI;−7.5 to 7.8), 73.0 % HS (−1.4; 95 % CI,−8.7 to 6.0), and 74.4 % NS (*p* = 0.91). The rate of organ failure or nosocomial infections in patients treated with NS was higher than those in the HS and HSD groups, but these differences were not statistically significant. The authors concluded that initial resuscitation with hypertonic solution in a pre-hospital setting did not improve 28 day survival. Future studies are warranted to better define the use of these fluids in a pre-hospital setting [42].

The Colloids versus Crystalloids for the Resuscitation of the Critically Ill (CRISTAL) trial enrolled 2857 intensive care unit patients admitted for hypovolemic shock to evaluate a possible decrease in mortality for the use of fluid resuscitation with colloids (*n* = 1443) versus crystalloids (1414). No difference in 28 day mortality was noted, but 90 day mortality improved in those resuscitated with colloids (30.7 % vs. 34.2 %). Patients receiving colloids also recorded more days free of vasopressor therapy and mechanical ventilation at 7 and 28 days, respectively. The incidence of organ failure did not differ between groups; however, the population of this study was heterogeneous (sepsis, trauma, others cases of hypovolemic shock) and differed from the previously discussed trials, which exclusively examined traumatic hemorrhagic shock patients. Patients in the crystalloid and colloid groups mainly received isotonic solutions (96 %) and hydroxylethyl starch (69 %), respectively. Subgroup analysis was not performed to compare hypertonic saline resuscitation versus isotonic crystalloids or colloids [43].

## Discussion

Bolus administration of HS is safe and effective and improved hemodynamic states from hemorrhagic hypotensive states [29–31]. In clinical studies, it has been shown that a bolus of 4 ml/kg of 7.5 % HS can enhance plasma sodium and osmolality from 147 to 154 mEq/l and 10 to 20 mOsm/kg, respectively; however, the increase in osmolality is temporary and starts to decline after 30 to 60 min [38–41]. This temporary increase in plasma osmolality can affect some functions of the immune system, which can persist for up to 24 h after HS administration [10, 42].

The immunomodulatory effects of hypertonic saline have clinical significance and include a reduction in the incidence of acute lung injury and infectious complications following hemorrhagic shock. Animal studies have shown that HS decreased acute lung injury and improved survival [5–8]; however, evidence that HS benefits survival in hemorrhagic shock patients is inconclusive.

The timing of HS administration appears to be critical. It has been shown that delayed administration of HS exacerbates the inflammatory response and tissue damage following trauma [44, 45]. A septic shock model study showed that the beneficial effects of HS diminished if it is administered after isotonic resuscitation [46]. The results of experimental and clinical studies indicate that the best time to administer HS to prevent inflammatory side effects is early during resuscitation in a pre-hospital setting. The results of pre-hospital resuscitation in clinical studies showed no significant effect on survival or improvement in outcome.

Several reasons for the disagreement between results from animal studies and clinical settings have been proposed. First, the amount and duration of HS infusion used in clinical studies could have been insufficient to decrease the inflammatory response in humans. Although the experimental studies in human showed anti-inflammatory effects of HS [10, 13], because of low sample size of these studies, it could not be concluded that HS can produce clinically relevant anti-inflammatory effects in every traumatic patient.

Another reason for disagreement may be the difference in model design. Animals were resuscitated using either HS or isotonic crystalloids immediately after blood withdrawal [5–8]. In clinical studies under real conditions, resuscitation may be delayed and isotonic fluid administration continued after hypertonic treatment. Evidence suggests that fluid resuscitation with isotonic solutions potentiate neutrophil activation associated with increased organ damage [11, 47, 48]. It has also been demonstrated that hypervolemia alone or massive resuscitation in trauma patients increases the risk of ARDS and mortality [49, 50]. It appears that isotonic resuscitation following HS administration attenuates the protective effects of HS on tissue injury and subsequent MODS.

Another matter for debate is whether trauma patients included in clinical studies actually had experienced IRI or had severe inflammatory conditions for which HS could be beneficial because HS only benefits a subpopulation who require more blood transfusions, have been severely injured, or require surgery. In these populations, severe inflammatory processes are generated in response to trauma. In these situations, the anti-inflammatory effect of hypertonic saline could be apparent.

Some patients could have existing metabolic acidosis at the time of treatment. Crystalloid fluids containing high chloride ion concentrations cause hyperchloremic metabolic acidosis [51]. Metabolic acidosis caused by lactate accumulation gradually disappears in hemorrhagic shock patients if tissue perfusion is restored, but the effect of HS resuscitation on acid–base status is not easily predictable. Moon et al. [52] showed that HS infusion in hemorrhagic shock models produces immediate and transient metabolic acidosis by increasing the plasma chloride concentration relative to the plasma sodium concentration. Metabolic acidosis affects some aspects of the immune system, increasing neutrophil phagocytosis, intracellular death, cytokines, ROS production, complement activation, and impairing leukocyte chemotaxis [53]. Metabolic acidosis is also associated with gut barrier dysfunction and may increase oxidative stress [54]. It is likely that hyperchloremic acidosis caused by administration of HS increases acidosis in already acidotic patients. Acidic conditions, particularly hyperchloremic acidosis, inhibit or weaken the immunomodulatory effects of HS and must be considered at the time of treatment.

## Conclusion

Hypertonic saline is safe when used as a resuscitation fluid in the early phase of trauma/hemorrhagic shock. Animal studies have demonstrated some benefit for survival rates, but clinical trials have failed to support such results in humans. HS has been advantageous for patients experiencing severe injury and requiring massive blood transfusions or surgery and in patients with TBI. Evidence suggests that these patients might experience a significant inflammatory response which can be ameliorated by the anti-inflammatory effects of HS.

The present study suggests that further clinical trials with large sample sizes be undertaken to clarify which subgroup of trauma/shock patents gain the most advantage from HS resuscitation. New clinical trials should be designed to better quantify the clinical benefits of continuing resuscitation after administration of the first HS bolus. It is also necessary to consider the acid–base status of a patient before administration of HS solution because the high chloride content of HS may worsen pre-existing lactic acidosis from superimposed hyperchloremic acidosis.

## Abbreviations

HS: Hypertonic saline
HSD: Hypertonic saline-dextran
MODS: Multiple organ dysfunction syndrome
IRI: Ischemia reperfusion injury
ROS: Reactive oxygen species
TBI: Traumatic brain injury
EC: Endothelial cell
MTOS: Major trauma outcome study
MAP: Mean arterial pressure
HR: Heart rate
SBP: Systolic blood pressure
ARDS: Acute respiratory distress syndrome
RCT: Randomized clinical trials
LR: Lactated ringer’s
NS: Normal saline
ROC: Resuscitation outcomes consortium
CRISTAL: Colloids versus crystalloids for the resuscitation of the critically ill
